# Multimorbidity Patterns Among Older Women Participants of the All of Us Research Program

**DOI:** 10.1093/geroni/igaf122.4345

**Published:** 2025-12-31

**Authors:** Mayra Tisminetzky, Mara Epstein, Yanhua Zhou

**Affiliations:** University of Massachusetts Chan Medical School, shrewsbury, Massachusetts, United States; univ of Massachusetts medical school, worcester, Massachusetts, United States; univ of Massachusetts medical school, worcester, Massachusetts, United States

## Abstract

Despite the fact that older women typically outlive men in industrialized countries and experience a higher prevalence of multimorbidity (the presence of two or more chronic conditions) with advancing age, limited knowledge exists regarding the specific patterns of chronic conditions that affect the lives of older women. We aimed to identify the prevalence and patterns of multimorbidity in older women (aged ≥65 years). Our study sample included 56,220 women aged 65 or older at study enrollment, who completed the three baseline surveys for the All of Us Research Program (AoURP). Overall mean age of this study population was 71 years; 71% were white and 9.7% were Hispanic. Overall, the five most common chronic conditions among AoURP participants were hyperlipidemia, hypertension, osteoarthritis, obesity and chronic pain (65%,62%, 61%, 47% and 42% prevalence, respectively). The top five pairs of co-occurring chronic conditions were: hyperlipidemia + hypertension; osteoarthritis + hyperlipidemia; osteoarthritis +hypertension; chronic pain + osteoarthritis; obesity + hypertension (51%,49%, 46%, 36% and 35% prevalence, respectively). More than 80% of study participants presented with two or more chronic conditions. Our findings also suggested that 25% of the older women participants of the AoURP reported having issues to complete daily activities and 1 out of 5 women reported having poor health. Given the high prevalence of chronic conditions and growing health-care needs within this aging population, understanding the distribution of specific multimorbidity combinations is of particular importance to optimize care delivery, reduce disparities, and address unmet healthcare needs.

